# The status of HBV infection influences metastatic pattern and survival in Chinese patients with pancreatic cancer

**DOI:** 10.1186/1479-5876-11-249

**Published:** 2013-10-08

**Authors:** Xiao-li Wei, Miao-zhen Qiu, Wei-wei Chen, Ying Jin, Chao Ren, Feng Wang, Hui-yan Luo, Zhi-qiang Wang, Dong-sheng Zhang, Feng-hua Wang, Yu-hong Li, Rui-hua Xu

**Affiliations:** 1 State Key Laboratory of Oncology in South China, Department of Medical Oncology, Collaborative Innovation Center of Cancer Medicine, Sun Yat-sen University Cancer Center, Guangzhou 510060, China

**Keywords:** Hepatitis B virus, Pancreatic cancer, Liver metastasis, Survival

## Abstract

**Background:**

It has been proved that hepatitis B virus (HBV) infection alters the metastatic pattern and affects survival in colorectal cancer (CRC) and hepatocellular carcinoma (HCC), while the influence of HBV infection on metastatic pattern and survival in patients with pancreatic cancer (PC) has not been investigated yet.

**Methods:**

We conducted an investigation to evaluate the impact of HBV infection on metastatic pattern and overall survival in PC. We collected the data of 460 PC patients treated in our hospital from 1999 to 2010. Serum HBV markers were tested with enzyme-linked immunosorbent assay. The impact of HBV infection on metastatic pattern and overall survival was analyzed.

**Results:**

We found that the incidence of synchronous liver metastasis was significantly higher in patients with HBsAg positive than those with HBsAg negative (46.0% vs 32.0%, P < 0.05), and higher in chronic HBV infection (CHB) group than both non HBV infection and resolved HBV infection group (61.1% vs 33.9%, P < 0.05, and 61.1% vs 28.7%, P < 0.05, respectively). What’s more, Kaplan-Meier analysis showed that CHB, resolved HBV infection and non HBV infection group had significant longer overall survival (OS) compared with inactive HBsAg carriers (IC) group (P=0.037, P=0.009, and P=0.019 respectively). But, in the multivariate analysis, only the CHB and non HBV infection group had significant better overall survival compared with IC group (P=0.010 and P=0.018 respectively).

**Conclusions:**

Our study found that HBV infection increased synchronous liver metastasis rate, and HBV infection status was an independent prognostic factor in PC patients.

## Background

Pancreatic cancer (PC) is one of the most aggressive diseases among digestive system malignancies. With both high incidence and mortality rate, it is responsible for the fourth leading cause of cancer in the USA [1]. Though less common in developing countries, the mortality rate of PC has been keeping increasing in the past decades and reached the sixth place among all malignancies in China [2]. Radical surgery, which is the only possible way to cure the disease, can be conducted in only about 20% patients. Approximately 60% patients are found to have metastases at the time of diagnosis [3]. Palliative treatments, mainly chemotherapy, radiotherapy, interventional therapy and immunotherapy, contribute limited survival benefit.

The prevalence of hepatitis B virus (HBV) infection is common worldwide, while the infection rate is especially high in some developing countries, including China [4]. It used to be one of the major public health problems in China, where 58.2% of the population had ever suffered from the infection of HBV in the 1980s. Since the introduction of universal infant immunization policy, the HBV carrier rate had dropped from 9.75% in 1992 to 7.18% in 2006 among the general Chinese population [5]. HBV infection has been demonstrated to play an important role in the development of hepatocellular carcinoma (HCC) and cholangiocarcinoma [6,7]. In addition, there have been increasing reports about the existence of HBV in extrahepatic tissues, such as pancreas, kidneys, skin, bone marrow, colon, lymph nodes, and vessel walls [8,9], with the manifestation of various severities of extrahepatic symptoms [10]. Moreover, there’re also investigations indicating that HBV infection is associated with a higher risk of some extrahepatic malignancies, including PC and non-Hodgkin’s lymphoma [11-15]. Some studies further presented a hypothetical mechanism of the development of PC with HBV infection: a multistep process from pre-neoplastic pancreatic lesions to invasive cancer resulted from persistent inflammation in the pancreas caused by HBV infection [16].

There are some reports available regarding the influence of HBV infection on metastatic pattern of colorectal cancer (CRC). Interestingly, compared with patients not infected with HBV, hepatic metastasis rate was shown to be decreased while the extrahepatic metastasis rate elevated in patient infected with HBV [17,18], and in HCC with HBV infection, multiple occurrences happened more frequently than intrahepatic metastasis [19], suggesting HBV infection might play an important role in the progression of some extrahepatic malignancies. In addition, studies showed that HBV infection also affected the survival of HCC and CRC patients [17,20]. However, the investigation about the impact of HBV infection on metastatic pattern is still blank, and the influence of different HBV infection status on survival in PC has not been fully studied. Thus, we embarked on this study to evaluate whether HBV infection status was associated with metastatic pattern and survival in PC.

## Materials and methods

### Ethics statement

All patients provided written informed consent for their information to be stored and used in the hospital database. Study approval was obtained from independent ethics committees at Cancer Center of Sun Yat-Sen University. The study was undertaken in accordance with the ethical standards of the World Medical Association Declaration of Helsinki.

### Study population

Based on the discharge diagnosis, Data of Patients treated at Sun Yat-sen University Cancer Center in Guangzhou, China from February 28, 1999 to March 31, 2011 who were diagnosed with pancreatic adenocarcinoma were retrospectively collected. 460 histopathologically confirmed cases with available test results for HBV infection were enrolled in the final analysis. 442 cases were excluded from the study since they were diagnosed with other types of pancreatic cancer, had no record of pathologic or imaging results. According to the HBV infection status at diagnosis, patients were categorized into four subgroups. The categorization of the groups was performed based on the clinical significance of each test index. Chronic hepatitis B (CHB), which stood for a status of active HBV replication, was characterized by HBsAg positive and at least one of HBeAg and HBV-DNA positive. Inactive HBsAg carriers (IC), meaning current HBV infection but without active replication, were defined as HBsAg positive, and both HBeAg and HBV-DNA negative. Resolved HBV infection, which indicated previous HBV infection but the virus was already eliminated, was featured by HBsAg negative and at least one of anti-HBe and anti-HBc positive. Non HBV infection, which meant no history of HBV infection judging from the serum test result, and referred to HBsAg, HBeAg, HBV-DNA, anti-HBe and anti-HBc negative, regardless of the status of anti-HBs [21,22]. A further categorization organize patients into two groups, HBsAg positive group (CHB+IC), and HBsAg negative group (resolved HBV infection +non HBV infection). Additionally, Informed consent to receive a test for HBV infection was obtained at the first visit of all patients, and the research was approved by the Ethics Committee of Sun Yat-sen University Cancer Center.

### Serologic assay for HBV infection

Blood samples were collected at the first visit of all the patients, and serum samples were separated for the test of HBV infection. Enzyme-linked immunosorbent assay was applied to the tests of hepatitis B surface antigen (HBsAg), hepatitis B surface antibody (anti-HBs), hepatitis B e antigen (HBeAg), hepatitis B e antibody (anti-HBe) and hepatitis B core antibody (anti-HBc). Polymerase chain reaction was used to detect HBV deoxyribonucleic acid (HBV-DNA). The quality of the tests was controlled by timeliness of the tests, and qualified positive and negative control serum.

### Assessment of metastases, treatment and follow-up of patients

All patients received chest, abdominal and pelvic computed tomography (CT) or magnetic resonance imaging (MRI) before surgery or chemotherapy, and the metastatic status were evaluated by imaging specialists. For those who underwent surgery, additional evaluations were given by surgeons. Thus accurate information with regard to synchronous liver and extrahepatic metastasis was obtained. Therapeutic and follow-up schedules were established and implied referring to the NCCN Clinical Practice Guidelines of pancreatic cancer.

### Statistical analysis

The statistical analyses were performed with SPSS for Windows V.13.0. A two tailed p value <0.05 was considered statistically significant. Differences of baseline parameters between HBsAg positive and negative group were evaluated by chi-square test, Student *t* test or Kruskal-Wallis H test based on the type of the data and comparison. Overall survival (OS) was the time interval from the date of diagnosis to death from PC or to the last date of follow-up. Overall survival (OS) curves were plotted with the Kaplan-Meier method, and differences were compared with log-rank test. A Cox regression was used for univariate and multivariate analysis. Hazard ratio (HR) and 95% confidence interval (95% CI) were computed with the Cox proportional-hazards model. Variables significantly prognostic in the univariate analysis were selected for the multivariable analysis using the forward stepwise method.

## Results

### Baseline characteristics

A total of 460 cases were qualified for the analyses. Among them, CHB, which means active HBV replication, was identified in 18 patients (3.9%). IC, which indicates HBV in the body without active replication, was identified in 45 patients (9.8%). resolved HBV infection, which represents previous HBV infection but the viruses have already been eliminated, was identified in 143 patients (31.1%). non HBV infection, which is the mark of the no history of HBV infection status, was identified in 254 patients (55.2%). Thus, 63 (13.7%) patients were enrolled in HBsAg positive group (CHB +IC), and 397 (86.3%) patients were classified into HBsAg negative group (resolved HBV infection + non HBV infection). The comparisons of baseline characteristics were listed in Table 1. No significant difference was found between HBsAg positive and negative group in age, tumor stage, tumor size, operation status and chemotherapy rate (Table 1). While the HBsAg positive group manifested with a significant male and young-aged predominance compared with the HBsAg negative group.

**Table 1:** Comparison of baseline characteristics between HBsAg positive and HBsAg negative group

**Variable**	**HBsAg positive group n (%)**	**HBsAg negative group n (%)**	**P**
No. of patients	63	397	
Gender	63(100)	397(100)	**0.04**
Male	50(79.4)	262(66.0)	
Female	13(20.6)	135(34.0)	
Age (yr)	63(100)	397(100)	**0.002**
Mean	52.89	57.82	
Median ± standard deviation	52±12.11	58±11.37	
Range	31-78	15-83	
TNM stage (AJCC)	63(100)	397(100)	0.08
Ia + Ib	1(1.6)	5(1.3)	
IIa + IIb	11(17.5)	106(26.7)	
III	12(19.0)	86(21.7)	
IV	39(61.9)	200(50.3)	
Tumor size (cm)	49(100)	298(100)	1.00
≤2	3(6.1)	20(6.7)	
>2	46(93.9)	278(93.3)	
Baseline CA19-9	59(100)	332(100)	0.36
Elevated	43(73.6)	260(79.9)	
Normal	16(26.4)	72(20.1)	
Operation	63(100)	397(100)	0.64
Yes	25(39.7)	170(42.8)	
No	38(60.3)	227(57.2)	
Chemotherapy	63(100)	397(100)	0.50
Yes	26(41.3)	182(45.8)	
No	37(58.7)	215(54.2)	

HBsAg positive group: chronic HBV infection (CHB) group + inactive HBsAg carriers (IC) group. HBsAg negative group: resolved HBV infection + non HBV infection group.

### Synchronous liver, extrahepatic and general metastasis

Synchronous liver metastasis was found in 156 (33.9%) patients. There were 11 (61.1%) cases of synchronous liver metastasis in CHB group, 18 (40.0%) cases in IC group, 41 (28.7%) cases in resolved HBV infection group, and 86 (33.9%) cases in non HBV infection group. Thus 29 (46.0%) were identified out of 63 cases in HBsAg positive group, and 127 (32.0%) out of 397 cases in HBsAg negative group, the incidence of synchronous liver metastasis was significantly higher in HBsAg positive group compared with HBsAg negative group (46.0% vs 32.0%, P = 0.03). As for extrahepatic metastasis, 8 cases were confirmed in HBsAg positive group and 72 in HBsAg negative group. There was no significant difference in the synchronous extrahepatic metastasis rate between the two groups (12.7% vs 18.1%, P = 0.29). In addition, no significant difference was found in general synchronous metastasis between HBsAg positive and negative group (55.6% vs 42.8%, P = 0.06). Detailed information was shown in Table 2.

**Table 2:** Influence of HBV infection on metastatic pattern in PC

**Compared groups**		**Liver metastasis n (%)**		**P value**	**Extrahepatic metastasis n (%)**		**P value**	**Metastasis n (%)**		**P value**
		**Yes**	**No**		**Yes**	**No**		**Yes**	**No**	
HBsAg positive group vs HBsAg negative group	HBsAg positive (63)	29 (46.0)	34 (54.0)	**0.03**	8 (12.7)	55 (87.3)	0.29	35 (55.6)	28 (44.4)	0.06
	HBsAg negative (397)	127 (32.0)	270 (68.0)		72 (18.1)	325 (81.9)		170 (42.8)	227 (57.2)	
HBV infection group vs non HBV infection group	CHB +IC +resolved HBV infection (206)	70 (34.0)	136 (66.0)	0.98	32 (15.5)	174 (84.5)	0.34	90 (43.7)	116 (56.3)	0.73
	Non HBV infection (254)	86 (33.9)	168 (66.1)		48 (18.9)	206 (81.1)		115 (45.3)	139 (54.7)	
Comparisons between HBV infection subgroups and non HBV infection group	CHB (18)	11 (61.1)	7 (38.9)	**0.02**	2 (11.1)	16 (88.9)	0.61	12 (66.7)	6 (33.3)	0.08
	Non HBV infection (254)	86 (33.9)	168 (66.1)		48 (18.9)	206 (81.1)		115 (45.3)	139 (54.7)	
	IC (45)	18 (40.0)	27 (60.0)	0.43	6 (13.3)	39 (86.7)	0.37	23 (51.1)	22 (48.9)	0.47
	Non HBV infection (254)	86 (33.9)	168 (66.1)		48 (18.9)	206 (81.1)		115 (45.3)	139 (54.7)	
	Resolved HBV infection (143)	41 (28.7)	102 (71.3)	0.29	24 (16.8)	119 (83.2)	0.60	55 (38.5)	88 (61.5)	0.19
	Non HBV infection (254)	86 (33.9)	168 (66.1)		48 (18.9)	206 (81.1)		115 (45.3)	139 (54.7)	
Comparisons among HBV infection subgroups	CHB (18)	11 (61.1)	7 (38.9)	0.13	2 (11.1)	16 (88.9)	1.00	12 (66.7)	6 (33.3)	0.26
	IC (45)	18 (40.0)	27 (60.0)		6 (13.3)	39 (86.7)		23 (51.1)	22 (48.9)	
	Resolved HBV infection (143)	41 (28.7)	102 (71.3)	0.15	24 (16.8)	119 (83.2)	0.58	55 (38.5)	88 (61.5)	0.13
	IC (45)	18 (40.0)	27 (60.0)		6 (13.3)	39 (86.7)		23 (51.1)	22 (48.9)	
	CHB (18)	11 (61.1)	7 (38.9)	**0.01**	2 (7.7)	16 (92.3)	0.78	12 (66.7)	6 (33.3)	**0.02**
	Resolved HBV infection (143)	41 (28.7)	102 (71.3)		24 (11.9)	119 (88.1)		55 (38.5)	88 (61.5)	

*CHB*, chronic HBV infection, *IC*, inactive carriers.

We further explored the influence of HBV infection on synchronous metastatic pattern in PC by classifying cases into more subgroups for comparisons (Table 2). No significant difference was identified between patients with the history of HBV infection (CHB + IC + resolved HBV infection) and those without (non HBV infection). Among the Comparisons between HBV infection subgroups (CHB, IC, and resolved HBV infection) and non HBV infection group, and comparisons among HBV infection subgroups, the synchronous liver metastasis rate was significantly higher in CHB group than non HBV infection or resolved HBV infection group (61.1% vs 33.9%, P = 0.02, and 61.1% vs 28.7%, P = 0.01), and the incidence of general metastasis in CHB group was significantly more frequent compared with resolved HBV infection group (66.7% vs 38.5%, P = 0.02). Because no significant difference was found in extrahepatic metastasis between CHB and resolved HBV infection group, the difference showed in general metastasis rate might be explained by increased liver metastasis, and has nothing to do with extrahepatic metastasis. In addition, none of the comparisons in synchronous extrahepatic metastasis reached significant difference.

### Follow-up and survival

**Figure 1:**
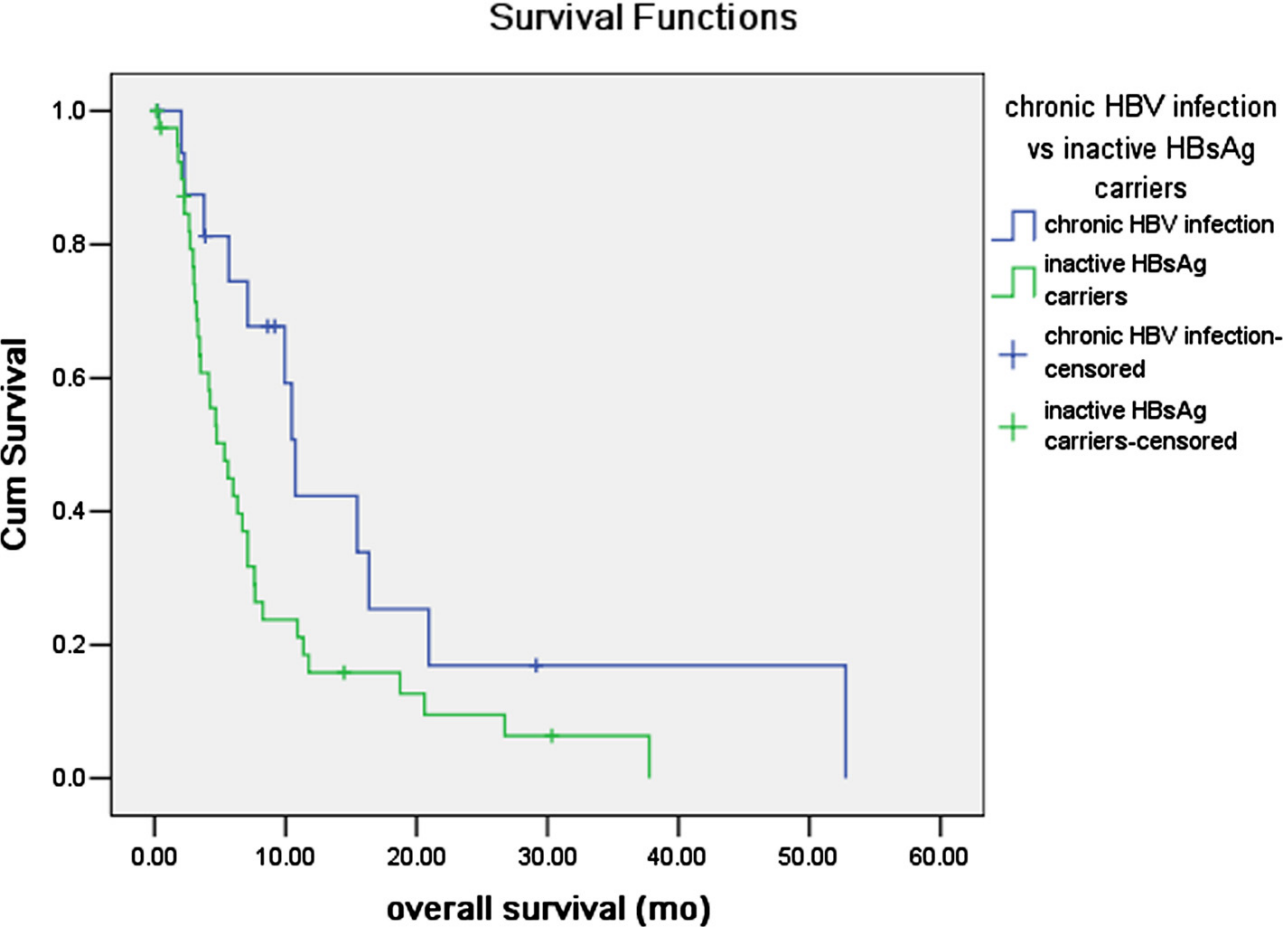
**Comparison of overall survival between chronic HBV infection (CHB) and inactive HBsAg carriers (IC) group.** In the Kaplan-Meier analysis compared with log-rank test, CHB group had a significant longer overall survival than IC group (P=0.037).

**Figure 2:**
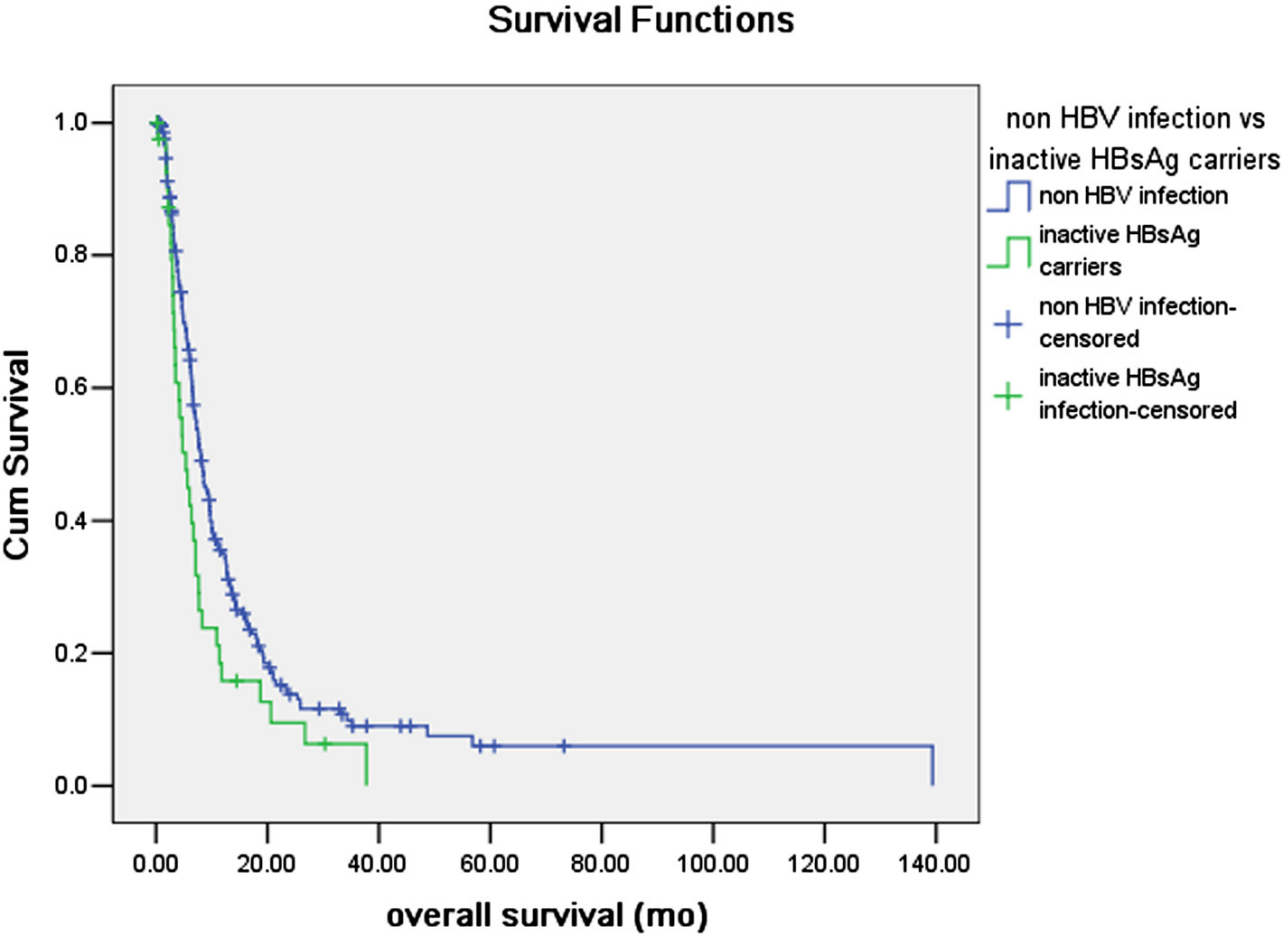
**Comparison of overall survival between non HBV infection and inactive HBsAg carriers (IC) group.** In the Kaplan-Meier analysis compared with log-rank test, non HBV infection group had a significant longer overall survival than IC group (P=0.019).

The mean and median follow-up time after discharge from hospital were 11.4 months and 6.5 months respectively, ranging within 0.1-139.4 months. 17 cases out of 18 cases in CHB group, 41 cases out of 45 cases in IC group, 123 cases out of 143 cases in resolved HBV infection group and 217 cases out of 254 cases in non HBV infection group were available of overall survival information, and were qualified for survival analysis compared with the log-rank test. The estimated general median OS time of PC was 7.6 months. In the univariate analysis exploring the influence of HBV infection status on OS in PC (Table 3), CHB, resolved HBV infection and non HBV infection group had significantly longer OS compared with IC group (P=0.037, P=0.009, and P=0.019 respectively). While, in the multivariate analysis adjusted by variables significantly prognostic in univariate analysis, only the CHB and non HBV infection group had significant better overall survival compared with IC group (P=0.010 and P=0.018 respectively, Table 4). The survival curves of CHB vs IC, resolved HBV infection vs IC and non HBV infection group vs IC plotted with Kaplan-Meier method were presented in Figure 1 and Figure 2.

**Table 3:** Association between prognostic factors, HBV infection and overall survival in a univariate analysis

**Factors**	**Number**	**Overall survival**	
		**Hazard ratio (95% confidence interval)**	**P value**
Age (≤ 70 yr/> 70 yr)	347/51	1.399 (1.015-1.927)	**0.039**
Gender (Male/Female)	268/130	0.822 (0.644-1.051)	0.117
Pretherapeutic weight loss (No/Yes)	150/248	1.282 (1.014-1.620)	**0.037**
The 7th tumor-node-metastasis (TNM) staging (AJCC) (Ia + Ib/IIa + IIb/III/IV)	5/100/86/207	1.339 (1.169-1.534)	**< 0.001**
Degree of differentiation (Poorly differentiated or mucinous adenocarcinoma/Moderate differentiated/Well differentiated)	224/130/44	0.666 (0.540-0.822)	**< 0.001**
Surgery (No/Yes)	228/170	0.522 (0.413-0.660)	**< 0.001**
Surgery (radical surgery R0 resection/radical surgery R1 resection/radical surgery R2 resection or Bypass or stent only or exploration or none)	54/4/112	1.410 (1.151-1.727)	**0.001**
Chemotherapy (No/Yes)	219/179	0.694 (0.552-0.873)	**0.002**
Baseline CA199 (Normal/Elevated)	80/297	1.614 (1.208-2.156)	**0.001**
Body mass index (≤ 25/> 25)	362/36	0.802 (0.533-1.209)	0.291
Smoking (No/Yes)	270/126	1.003 (0.791-1.272)	0.981
Alcohol consumption (No/Yes)	315/80	1.183 (0.901-1.552)	0.225
HBV infection			
HBsAg positive/HBsAg negative	58/340	1.285 (0.943-1.749)	0.110
Non HBV infection/CHB +IC +resolved HBV infection	217/181	1.010 (0.807-1.266)	0.928
Non HBV infection/CHB	217/17	0.776 (0.432-1.394)	0.394
Non HBV infection/IC	217/41	1.538 (1.072-2.208)	**0.019**
Non HBV infection/RHB	217/123	0.923 (0.714-1.193)	0.541
CHB/IC	17/41	2.060 (1.044-4.064)	**0.037**
Resolved HBV infection/IC	123/41	1.681 (1.137-2.486)	**0.009**
Resolved HBV infection/CHB	123/17	0.862 (0.471-1.578)	0.629

Hazard ratios and 95% confidence intervals were calculated with the Cox proportional-hazards model. P value was worked out by Kaplan-Meier method with log-rank test.

*CHB*, chronic HBV infection, *IC*, inactive carriers.

**Table 4:** Association between prognostic factors, HBV infection and overall survival in a multivariate analysis

**Factors**	** Overall survival**	
	**Hazard ratio (95% confidence interval)**	**P value**
Age (≤ 70 yr/> 70 yr)	1.527 (1.075-2.169)	**0.018**
The 7th tumor-node-metastasis (TNM) staging (AJCC) (Ia + Ib/IIa + IIb/III/IV)	1.310 (1.129-1.520)	**< 0.001**
Degree of differentiation (Poorly differentiated or mucinous adenocarcinoma/Moderate differentiated/Well differentiated)	2.134 (1.729-4.538)	**0.031**
Surgery (No/Yes)	0.553 (0.429-0.713)	**< 0.001**
Chemotherapy (No/Yes)	0.648 (0.506-0.829)	**0.001**
Baseline CA199 (Normal/Elevated)	1.602 (1.179-2.176)	**0.003**
HBV infection		**0.038**
IC	1 (reference)	
CHB	0.414 (0.213-0.807)	**0.010**
Resolved HBV infection	0.683 (0.450-1.036)	0.073
Non HBV infection	0.630 (0.429-0.925)	**0.018**

*CHB*, chronic HBV infection, *IC*, inactive carriers.

## Discussion

Our research found that the incidence of synchronous liver metastasis of pancreatic cancer (PC) was higher in patients with HBsAg positive than those negative, while no significant difference of extrahepatic metastasis was found between the two groups. Compared with non HBV infection group, only chronic hepatitis B (CHB) group was demonstrated to have significant higher rate of liver metastasis, which indicated that HBV infection with active replication had the greatest influence on liver metastasis of PC. What’s more, CHB and non HBV infection group showed significant better overall survival than IC group, indicating HBV infection status to be an independent prognostic factor in PC patients.

There were mounts of studies investigating the impact of HBV infection on metastatic patterns of other malignancies. Incidence of liver metastasis was demonstrated to be significantly decreased, and extrahepatic metastasis significantly increased in colorectal cancer (CRC) patients with HBV infection than non-infection ones, and significance of the difference existed in all status of HBV infection, including CHB, IC, and resolved HBV infection [18]. This impact had nothing to do with coexisting liver injury [17]. In hepatocellular carcinoma (HCC) with HBV infection, multiple occurrences happened more frequently than intrahepatic metastasis [19]. To date, our study was the first report about influence of HBV infection on metastatic pattern of PC. We found that compared with non HBV infection group, only chronic HBV infection group (CHB) showed a significant higher liver metastasis rate. Thus, it can be concluded that the impacts of HBV infection on liver metastasis vary in different malignancies. Tumor progression was pushed by intrinsic mutations and influenced by the microenvironment [23]. Although before tumor cell implantation, the microenvironment in the liver might share much similarity, the differences of tumor intrinsic properties and the microenvironment nurtured by the interaction between tumor cells and surroundings after preliminary implantation would result in discriminated outcomes. Besides, HBV infection had been proved to be one of the risk factors in PC [11,12], and HBV DNA and antigens were identified in PC tissues [24]. Moreover, both liver and pancreas progenitors had the same origin of the endoderm cells in the embryonic foregut [25,26], and the two organs shared the same conduct for external secretion and blood vessels. It was reasonable to postulate that the current understanding of the relationship between liver, pancreas, and HBV infection was only a tip of the iceberg. It was also reasonable to assume that developed from an HBV infected microenvironment with persistent inflammation, PC cells might have a predilection for the analogous hepatic nest infected with HBV. Furthermore, HBV proteins were able to trigger the self-maintaining and amplifying looped generation of certain cytokines in liver, such as prostaglandin E2 (PGE2) and growth factors, which activated cell signal pathways, and promoted growth of cancer cells [27,28]. While current study concentrating on the correlation between HBV and PC were mainly restricted in epidemiologic investigation and carcinogenesis mechanism, regarding HBV infection as a risk factor of PC, perhaps further attention should also be paid to understand how HBV infection affects PC progression.

HBV infection was proved to have more or less influence on survival in some malignancies. Hatanaka K, et al. found the survival of HCC patients infected with HBV to be the poorest compared with patients infected with HCV or non HBV/HCV [20]. In terms of CRC, previous studies didn’t reach consistent conclusion about the survival impact of HBV infection [17,18,29]. Concerning the impact of HBV infection on PC in our study, both CHB and non HBV infection group had significantly longer OS compared with IC group in multivariate analysis.

It was interesting that different HBV infection status showed differences in the overall survival in PC patients. The results that patients infected with actively replicative HBV (CHB group) showed better survival than inactive HBsAg carriers (IC group), and patients classified into resolved HBV infection had no significant OS difference compared with inactive HBsAg carriers (IC group) might seem to be somewhat confusing. Here we listed some explanation for the results of survival analysis in our research.

Firstly, HBV antigens might influence the overall survival in PC patients. Some previous studies gave us clues. HBsAg, which existed in CHB and IC groups, was found to down-regulate the production of interleukin-12 (IL-12), a cytokine which was identified to possess the anti-cancer power [30]. However, the expression of HBeAg in liver cells was demonstrated to be associated with more serious liver cell damage, and enhanced production of inferferon (IFN)-gamma [31,32]. Considering the HBeAg expression was also demonstrated to exist in PC tissues with HBV infection [24], such mechanism might likewise contribute to anti-pancreatic cancer cell activity. These findings partly explained why CHB group (with both HBeAg and HBsAg positive) showed better survival than IC group (with HBsAg positive and HBeAg negative), although patients in CHB group had active HBV proliferation, which might bring liver dysfunction and make patients’ condition worse. It could also partly explain why IC group showed worse survival than non HBV infection group.

Secondly, starting from the time they received chemotherapy, patients with active HBV infection in our center were routinely given anti-HBV therapy to control the activation of HBV and prevent the outbreak of hepatitis B virus. Anti-HBV drugs were shown to have immunoregulatory effects [33] and might also influence the anti-cancer effectiveness of the immune system.

Thirdly, HBV was hepatotrophic virus and active HBV replication in liver cells triggered the immune response, mainly cellular immunity, which induced the death of liver cells and caused hepatic fibrosis. Because HBV was also demonstrated to be existed in PC tissues, such immune response acted on liver cells might also work on pancreatic cancer cells. Here we would like to share a case reported by Fujimoto, K, et al. They reported a case of complete remission of splenic marginal zone lymphoma after an acute flare-up of hepatitis B in a hepatitis B virus carrier [34]. There were studies finding HBV to be a risk factor for non-Hodgkin lymphoma, and HBV infection was also found to be existed in B-cell non-Hodgkin lymphoma tissues [35]. The case reported might be an extreme example of the possible mechanism we listed above to kill the cancer cells in which HBV replicate actively.

Last but not the least, patients in resolved HBV infection group and IC group showed no significant difference in OS. This conclusion was hard to explain since patients in resolved HBV group might possess a more effective immune system, thus shall have a better survival than patients in IC group. Because the P value (0.073) in the multivariate analysis of “resolved HBV group vs IC group” was quite close to the cut point of 0.05 we set and the sample size for survival analysis was relatively small (41 for IC group, and 123 for resoled HBV infection group), there was a possibility that our research didn’t reach the existed difference between the two groups. But, there were also possible small number of inactive HBV infection cases in the “resolved HBV infection” group we defined according to the serum test result, depending on the sensitivity of the serum test for HBV infection, and they might confound the result of survival analysis between the two groups.

To summarize, the mechanism of how different HBV infection status affect the progression of PC was still not clear, more researches about the clinical data and laboratory mechanism are both needed.

There were some limitations in our study. To start with, our data was retrospectively collected from medical records, considering inevitably missed information about progress free survival (PFS) and disease free survival (DFS) during follow up, we didn’t introduce PFS or DFS into analysis. In addition, we investigated the impact of HBV infection only on synchronous metastasis, owning to our concern about imprecision of the information about metachronous metastasis. However, the information about synchronous metastasis was qualified, because we collected it from the medical records of first visit of patients, for each of them, a chest, abdominal and pelvic CT or MRI were conducted and evaluated by imaging specialists, and those underwent surgery were further evaluated by surgeons during operations. What’s more, we enrolled PC patients in all tumor stages, which was an aggregation of PC patients with metastasis all along the disease progress, thus the result of our study was in line with the metastasis status of general PC population. Last but not least, although we collected cases from the time duration of more than 10 years, the number of cases in each group was still limited, more data and work are needed in future.

In conclusion, our study discovered that the incidence of liver metastasis in PC patients with HBsAg positive was higher than those with HBsAg negative. Compared with patients with no history of HBV infection and those with resolved HBV infection, chronic HBV infection promoted liver metastasis in PC. What’s more, patients with chronic HBV infection and non HBV infection had significantly better overall survival compared with inactive HBsAg carriers in pancreatic cancer.

## Conclusion

Our study was the first to find HBV infection increased liver metastasis rate and the status of HBV infection was an independent prognostic factor in pancreatic cancer. Since the existing studies investigating the relationship between HBV infection and pancreatic cancer mainly regarded HBV infection as a risk factor, our work suggested that more investigations should focus on the impact of HBV infection on progression in pancreatic cancer.

## Abbreviations

HBV: Hepatitis B virus; CRC: Colorectal cancer; HCC: Hepatocellular carcinoma; PC: Pancreatic cancer; HBsAg: Hepatitis B surface antigen; anti-HBs: Hepatitis B surface antibody; HBeAg: Hepatitis B e antigen; anti-HBe: Hepatitis B e antibody; anti-HBc: Hepatitis B core antibody; CHB: Chronic HBV infection; IC: Inactive HBsAg carriers; OS: Overall survival; DFS: Disease free survival; PFS: Progress free survival.

## Competing interests

We have no financial or personal relationships with other people or organizations that would bias our work. No benefits in any form have been received or will be received from a commercial party related directly or indirectly to the subject of our article. We declare that we have no competing interests.

## Authors’ contributions

XLW and MZQ participated in the clinical data collecting of the pancreatic cancer patients and drafted the manuscript. XLW and WWC performed the statistical analysis. YJ, CR, FHW and FW participated in the design of the study. HYL, ZQW, DSZ and YHL participated in the statistical analysis. RHX conceived of the study, participated in the design and coordination and helped to draft the manuscript. All authors read and approved the final manuscript.
